# An Aquaporin Gene (*KoPIP2;1*) Isolated from Mangrove Plant *Kandelia obovata* Had Enhanced Cold Tolerance of Transgenic *Arabidopsis thaliana*

**DOI:** 10.3390/bioengineering10070878

**Published:** 2023-07-24

**Authors:** Jiao Fei, Youshao Wang, Hao Cheng, Hui Wang, Meilin Wu, Fulin Sun, Cuici Sun

**Affiliations:** 1 State Key Laboratory of Tropical Oceanography, South China Sea Institute of Oceanology, Chinese Academy of Sciences, Guangzhou 510301, China; feijiao@scsio.ac.cn (J.F.); wanghui224@mails.ucas.ac.cn (H.W.); mlwu@scsio.ac.cn (M.W.); flsun@scsio.ac.cn (F.S.); scuici@scsio.ac.cn (C.S.); 2 Southern Marine Science and Engineering Guangdong Laboratory, Guangzhou 511458, China; 3 Innovation Academy of South China Sea Ecology and Environmental Engineering, Chinese Academy of Sciences, Guangzhou 510301, China

**Keywords:** aquaporin, cold stress, gene expression, *Kandelia obovata*, mangrove, transgenic

## Abstract

Aquaporins (AQPs) are essential channel proteins that play central roles in maintaining water homeostasis. Here, a novel aquaporin gene, named *KoPIP2;1*, was cloned from the mangrove plant *Kandelia obovata* by RACE technology. The *KoPIP2;1* gene was 1404 bp in length with an open reading frame (ORF) of 852 bp, encoded with 283 amino acids. Database comparisons revealed that *KoPIP2;1* protein shared the highest identity (91.26%) with the aquaporin *HbPIP2;2*, which was isolated from *Hevea brasiliensis.* Gene expression analysis revealed that the *KoPIP2;1* gene was induced higher in leaves than in stems and roots of *K. obovata* under cold stress. Transient expression of *KoPIP2;1* in *Nicotiana benthamiana* epidermal cells revealed that the *KoPIP2;1* protein was localized to the plasma membrane. Overexpressing *KoPIP2;1* in *Arabidopsis* significantly enhanced the lateral root number of the transgenic lines. *KoPIP2;1* transgenic *Arabidopsis* demonstrated better growth, elevated proline content, increased superoxide dismutase (SOD) and peroxidase (POD) activities, and reduced malondialdehyde (MDA) content compared with the wild-type *Arabidopsis* when exposed to cold stress. The findings suggest that overexpression of *KoPIP2;1* probably conferred cold tolerance of transgenic *Arabidopsis* by enhancing osmoregulation and antioxidant capacity. This present data presents a valuable gene resource that contributes to the advancement of our understanding of aquaporins and their potential application in enhancing plant stress tolerance.

## 1. Introduction

The process of water movement plays a crucial role in facilitating optimal growth and reproductive functions in plants. Aquaporins (AQPs), a group of water channel proteins, can effectively enhance the permeability of water [1]. In addition to facilitating plant growth and development, AQPs play crucial roles in various aspects of plant water relations, including the regulation of hydraulic processes in roots and leaves, maintenance of cell turgor pressure, facilitation of root water uptake, and promotion of leaf transpiration [2]. AQPs regulate the facilitated diffusion of water and other uncharged solutes, including glycerol, hydrogen peroxide, carbon dioxide, ammonia, small organic acids, urea and metalloids. They also play a crucial role in maintaining water status, osmotic regulation, signal transduction, detoxification processes as well as the acquisition transportation of nutrients [1,3,4,5]. As water transporters, AQPs are involved in plant response to multiple external stimuli, including cold, drought, salt and waterlogging stress [6,7]. AQPs are classified into five subfamilies based on sequence similarity and subcellular localization: plasma membrane intrinsic proteins (PIPs), tonoplast intrinsic proteins (TIPs), nodulin-26-like intrinsic proteins (NIPs), small basic intrinsic proteins (SIPs) and poorly characterized X intrinsic proteins (XIPs), of which PIPs are further subdivided into two phylogenetic subgroups: PIP1 and PIP2 [8,9]. All members of the AQP family contain highly conserved structural features and can form tetrameric quaternary structures in cell membranes [10]. Each AQP monomer contains six transmembrane domains (TM1–TM6), which are connected by five loops (loop A–loop E) [11]. The second structural characteristic is the aromatic/arginine (Ar/R) selectivity filter, which participates in substrate selection and prevents the ingress of other molecules into the aqueous pore [12]. The structural features of plant AQPs confer them with a wide range of selectivity for water and other solutes [2].

Studies have shown that some AQP genes can benefit plants under favorable and/or abiotic stress conditions [13,14], while others may have adverse effects [15,16]. However, our understanding of these proteins remains limited due to conflicting functions. Mangrove plants are important members of marine wetland ecosystems and mainly grow in the intertidal zone of tropical and subtropical regions [17]. An extraordinary cold or frost would significantly impede the growth of mangrove plants [18,19], and water imbalance is one of the main contributors to chilling injury [20]. The species *Kandelia obovata* exhibits the highest level of cold resistance among mangrove plants [21]. AQPs function as water channels and gated ion channels [22] and play important roles in water balance and water use efficiency [23]. Therefore, the AQPs of *K. obovata* may play positive roles in response to cold stress, and/or be useful genes for studying the stress response of mangroves. However, there is currently little literature about AQPs in *K. obovata* or other mangrove species [24,25,26,27].

In our previous study, we isolated an expressed sequence tag (EST) (Ko4002) from *K. obovata* under cold stress, which exhibited homology with other AQPs [28]. In order to gain a comprehensive understanding of the characteristics and functions of this gene, we conducted cloning and characterization of the full-length sequence of this AQP gene in this study, which was designated as *KoPIP2;1*. Furthermore, overexpression of *KoPIP2;1* in *Arabidopsis thaliana* was performed to test its function under cold stress. This study will provide valuable clues for the function of AQP genes in *K. obovata* under cold stress, and also help to improve our understanding of the mechanism of stress resistance in mangrove plants.

## 2. Materials and Methods

### 2.1. Plant Material, Growth Conditions and Treatments

The seeds of *K. obovata* were collected from Guangdong Mangrove Ecological Technology Co. LTD (Zhuhai, Guangdong, China) and sown in clean sand at room temperature. The 3 months seedlings were transferred into growth chamber with normal condition (25 °C, relative humidity 75%, 14 h light/10 h dark cycle). After 7 days, these seedlings were treated under cold treatment (5 °C, relative humidity 75%, 14 h light/10 h dark cycle) for 0 h, 6 h, 12 h, 1 d, 2 d, 4 d, 7 d, 15 d and 20 d. The control group consisted of seedlings treated at 5 °C for 0 d. The leaves, stems and roots of treated seedlings were individually collected and rapidly frozen in liquid nitrogen before being stored at −80 °C until further use.

### 2.2. Isolation of Total RNA and cDNA Synthesis

Total RNA was extracted from *K. obovata* leaves using the Tiangen RNA plant Plus Reagent (Tiangen Biotech, Beijing, China) following a modified version of the described method [29]. Before the chloroform extraction step, we added a step wherein we extracted samples with phenol/chloroform/isoamyl (25:24:1 of volume ratio). The subsequent steps followed the reagent operating instructions. The integrity and purity of total RNA were assessed using a Nanodrop 1000 spectrophotometer (Thermo Scientific, Wilmington, DE, USA) and confirmed by electrophoresis on a 1.0% agarose gel. The potential contamination of genomic DNA was eliminated through the use of RNase-free DNaseI (Promega, Madison, WI, USA). Subsequently, each RNA sample was equally mixed and subjected to SMART^TM^ reverse transcription Kit (Clontech, San Francisco, CA, USA) for synthesizing the first strand cDNA in accordance with the manufacturer’s instructions. The resulting cDNA samples were utilized to clone the full-length of the *KoPIP2;1* gene and to conduct the RT-qPCR analysis.

### 2.3. Cloning the Full-Length cDNA of KoPIP2;1 Gene

The partial sequence of the *KoPIP2;1* gene (GenBank accession number: JZ585815.1) has been mentioned in our previous study [28]. This EST sequence was used as the template for designing specific primers to clone the full-length cDNA sequence by rapid amplification of cDNA ends (RACE) technology in this study. The SMART RACE cDNA amplification kit (Clontech, San Francisco, CA, USA) was employed for PCR amplification according to the manufacturer’s protocol. Gene-specific primers (GSP1, 5′-GAGAGCCACAAAACAAAAGGAGGGGGT-3′, GSP2, 5′-ACCAGACCACAGCCACAGATCGCACCCA-3′) were designed for primary PCR amplification of the 5′ and 3′ end sequences of the *KoPIP2;1* gene. Nested PCR reactions were performed using nested primers (NGSP1, 5′-GACTACCATGACCCACCTCCTGCTCCCT-3′, NGSP2, 5′-GGCCGGGTTAATGTGTCCTCCAGAGATA-3′) to obtain DNA fragments and RACE products which were purified by agarose gel electrophoresis and confirmed by sequencing. The 3′- and 5′- nucleotide sequences obtained were assembled using DNAMAN 6.0 software to obtain the full-length sequence of *KoPIP2;1* with overlapping regions. Subsequently, primers were designed based on the assembled sequence for cloning the complete length of *KoPIP2;1*. The newly obtained *KoPIP2;1* sequence was then submitted to the company (BGI, Shenzhen, Guangdong, China) for sequencing, confirming the full-length cDNA sequence of *KoPIP2;1*, which was subsequently submitted to GenBank and assigned accession number KP267759.1.

### 2.4. Bioinformatics Analysis of KoPIP2;1 Gene

The deduced amino acid sequence of *KoPIP2;1* (GenBank accession number: KP267759.1) was predicted using the ORF Finder tool provided by the National Center for Biotechnology Information (NCBI) (https://www.ncbi.nlm.nih.gov/orffinder/, accessed on 24 June 2023). Sequence comparisons with known sequences were conducted utilizing the NCBI BLAST tools (http://blast.ncbi.nlm.nih.gov/Blast.cgi, accessed on 24 June 2023). The molecular weight and theoretical pI were predicted using the Compute pI/MW tool (http://web.expasy.org/compute_pi/, accessed on 24 June 2023), while TMHMM-2.0 (https://services.healthtech.dtu.dk/services/TMHMM-2.0/, accessed on 24 June 2023) was utilized to predict the transmembrane domain. Motif Scan (http://myhits.isb-sib.ch/cgi-bin/motif_scan, accessed on 24 June 2023) was employed to detect motif sequences, and subcellular localization was predicted by combining PSORT (http://www.psort.org/, accessed on 24 June 2023) and Softberry ProComp v. 9.0 (http://www.softberry.com/berry.phtml?topic=protcomppl&group=programs&subgroup=proloc, accessed on 24 June 2023). The ClustalX 1.83 software was utilized to perform multiple sequence alignments. A total of 40 homologues of AQPs from the NCBI database were employed to investigate the evolutionary relationship of the deduced *KoPIP2;1* protein. The phylogenetic tree was constructed using the Neighbor-Joining method in MEGA 5.0 software. The three-dimensional (3D) structure of *KoPIP2;1* was generated using the SWISS-MODEL tool (http://www.swissmodel.expasy.org/, accessed on 24 June 2023).

### 2.5. Expression Analysis by RT-qPCR

The transcriptional levels of the *KoPIP2;1* gene under cold stress were determined using a real-time quantitative PCR (RT-qPCR) method. Seedlings were subjected to 5 °C for different durations (0 h, 6 h, 12 h, 1 d, 2 d, 4 d, 7 d, 15 d and 20 d), with seedlings treated at 0 day serving as control. The leaves, stems and roots were harvested separately under different conditions. Total RNA was extracted from samples and cDNA synthesis was performed following the methods according to the above Section 2.2. The *K. obova*’s 18S rRNA was used as reference [30,31]. Each treatment was conducted with three independent biological replicates. The primers for *KoPIP2;1* (forward primer, CTCGGCGAAGGACTACCA; reverse primer, TACCCAGAATGTCAACACCAG) were designed for RT-qPCR analysis. The SYBR Premix Ex Taq^TM^ II (Takara, Dalian, Liaoning, China) reagents were used in the RT-qPCR experiment and analyzed by iCycler iQ5 real-time PCR detection system (Bio-Rad, Hercules, CA, USA). The cycling parameters included an initial denaturation at 95 °C for 30 s, followed by 40 cycles of denaturation at 95 °C for 5 s, annealing at 55 °C for 30 s and extension at 72 °C for 30 s. Each RT-qPCR reaction was performed in triplicate. Transcripts were quantified using 2^−∆∆CT^ method [32]. The data were expressed as mean ± standard deviation (x ± SD). Statistical analysis was conducted using the Student’s *t*-test, and graphical representation was generated with GraphPad Prism 7.0 (GraphPad Software, San Diego, CA, USA).

### 2.6. Subcellular Localization Analysis

The *KoPIP2;1* gene was cloned into the pFGC5941-35S-GFP vector [33] with N-terminal fusion using the Hieff Clone^®^ Plus One Step Cloning Kit (Yeasen Biotech, Shanghai, China) following the manufacturer’s instructions. Subsequently, the resulting fluorescence-tagged *35S-KoPIP2;1*-GFP vector was introduced into *Nicotiana benthamiana* epidermal cells via agrobacterium-mediated transformation. The preparation of *N. benthamiana* leaves and transformation procedures were carried out as previously described [28]. Briefly, recombinant plasmid 35S-*KoPIP2;1*-GFP was transiently introduced into the leaf epidermis of *N. benthamiana* via *Agrobacterium tumefaciens* EHA105-mediated transformation, while the vector containing only 35S-GFP served as a control. The fluorescence signal in leaves was visualized using Zeiss LSM710 laser scanning confocal microscopy (×63), with excitation and emission wavelengths of 489 nm and 510 nm for GFP signal detection, respectively. Fluorescence intensity was measured at 493 to 542 nm for GFP.

### 2.7. Generation of KoPIP2;1 Transgenic Arabidopsis Plants

The *KoPIP2;1* gene was cloned into the binary vector pGW505-1 using Invitrogen Company’s Gateway technology, following the manufacturer’s instructions, to investigate its function in a heterologous expression system. The primers TOPO-F (5′-GGGGACAAGTTTGTACAAAAAAGCAGGCTCGATGGCAAAGGACGTTGAAGTTCAAG-3′) and TOPO-R (5′-GGGGACCACTTTGTACAAGAAAGCTGGGTCAGCATTGCTCCTGAAGGATCCGA-3′) were designed for directional cloning in accordance with the specified requirements. Subsequently, the amplified PCR products were cloned into an entry vector using the pENTR™/D-TOPO™ cloning Kit (Invitrogen, Carlsbad, CA, USA) and subjected to sequencing analysis. Confirmed recombinant entry vectors were subsequently transferred into the plant binary vector pGWB505 through LR Clonase™ II Enzyme Mix (Invitrogen, Carlsbad, CA, USA) for construction of plant Gateway^®^ expression vector and sequenced. The pGWB505 vector containing *KoPIP2;1* recombinant clones was introduced into *A. tumefaciens* strain EHA105 using the freeze–thaw method for transformation. Positive colonies were then subjected to floral dip-mediated transformation in *Arabidopsis* plants [34]. Positive transgenic lines of *A. thaliana* were subsequently obtained and selected by culturing on MS medium agar plates supplemented with 50 mg/L kanamycin. The integration of transgenes in these plants was confirmed through RT-PCR (reverse transcription-PCR) analysis using gene-specific primers F2 (5′-ATGGCAAAGGACGTTGAAGTTCAAG-3′) and R2 (5′-TTAAGCATTGCTCCTGAAGGATCCG-3′). The transgenic lines were advanced through self-pollination until T3 transgenic plants were obtained. Finally, the T3 or T4 homozygous lines were utilized for functional analysis.

### 2.8. Physiological Analysis of Transgenic A. thaliana Lines Exposed to Cold Stress

Wild-type (WT) *A. thaliana* and transgenic *Arabidopsis* lines overexpressing *KoPIP2;1* (Lines 2, 3, and 5) were subjected to cold treatments. Cold stress was applied to 12-day-old seedlings by incubating them at 5 °C for 10 days. Seedlings grown under normal conditions at 22 °C were used as controls. The fresh weight and number of lateral roots in these seedlings were quantified. The levels of proline and malondialdehyde (MDA), as well as the activities of superoxide dismutase (SOD) and peroxidase (POD), were quantified using the corresponding assay kits (Jiancheng Bioengineering Institute, Nanjing, Jiangsu, China) following the manufacturer’s instructions. All experiments were conducted in triplicate. Statistical analysis was performed using GraphPad Prism 7.0 software (GraphPad Software, San Diego, CA, USA). One-way ANOVA followed by Duncan’s test was employed to determine significant differences using SPSS statistics 25.

## 3. Results

### 3.1. Characterization and Sequence Analysis of KoPIP2;1

**Figure 1:**
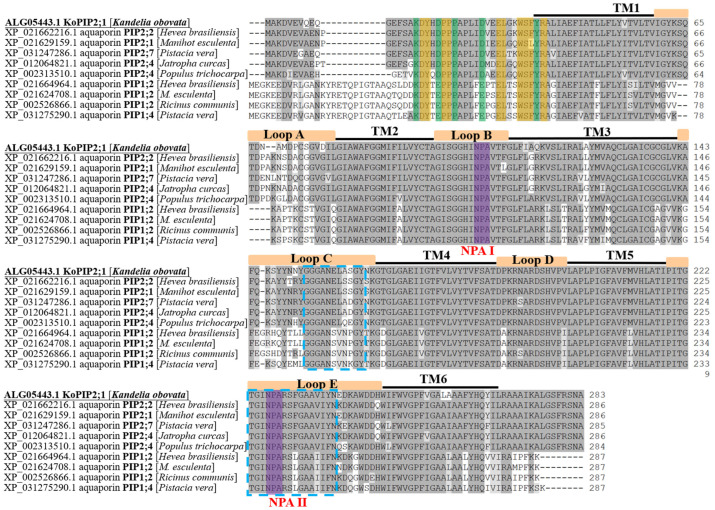
Sequence alignment of *KoPIP2;1* and other plant PIP1s and PIP2s. The amino acids that are identical among proteins are shaded in dark gray, while the similar acids are shaded in light gray. The conserved amino acids of PIPs at N-terminal (P/K)/DYX(E/D)PP(P/R)X_3-4_(E/D)XXELXXWSF(Y/W)R are highlighted with green and yellow shading. The six transmembrane domains (TM1–TM6) and five loops (Loop A–Loop E) of AQPs are represented by black lines and orange boxes above the sequences, respectively. The two conserved NPA motifs (NPA I and NPA II) are shaded in purple. The blue dotted boxed sequences depict the two conserved sequences (GGGANXXXXGY and TGI/TNPARSL/FGAAI/VI/VF/YN) present in all of the PIPs.

The full-length cDNA sequence of this aquaporin gene with 1404 base pairs (bp) was generated and named *KoPIP2;1* (GenBank ID: KP267759.1). The *KoPIP2;1* sequence comprises a 136 bp 5′-untranslated region (UTR), a 416 bp 3′-UTR, and an 852 bp complete open reading frame (ORF) that encodes a protein of 283 amino acids with a calculated molecular weight (MW) of 30.41 kDa and an isoelectric point (p*I*) of 6.05. Bioinformatics analysis revealed that the *KoPIP2;1* protein contains 24 positively charged residues (Arg and Lys) and 21 negatively charged residues (Asp and Glu) and is predicted to be localized in the plasma membrane. Similar to other PIPs, *KoPIP2;1* contains six transmembrane domains (TM1–TM6), five loops (Loop A–Loop E) and conserved dual NPA motifs (NPA Ⅰ and NPA Ⅱ) (Figure 1), which are typical domains of the AQP family. Additionally, the *KoPIP2;1* protein harbors two highly conversed sequences (signature GGGANXXXXGY and signature TGI/TNPARSL/FGAAI/VI/VF/YN in Loop C and Loop E, respectively) within its central region, as well as a distinctive conserved sequence (P/K)/DYX(E/D)PP(P/R)X_3-4_(E/D)XXELXXWSF(Y/W)R at its N-terminal, all of which are typical features of the PIP subfamily [35]. Furthermore, sequence analysis showed that *KoPIP2;1* exhibited the highest sequence identity (91.26%) with *Hevea brasiliensis* aquaporin *HbPIP2;2* (GenBank accession number: XP_021662216.1). Sequence alignment also demonstrated that *KoPIP2;1* displayed greater similarity to PIP2s than PIP1s in both the N-terminus and C-terminus regions (Figure 1). These findings suggest that *KoPIP2;1* belongs to the PIP2 family.

The SWISS-MODEL online tool was utilized to construct the 3D model of *KoPIP2;1* (Figure S1). A sequence similarity comparison between *KoPIP2;1* and its template (*SoPIP2-1*, *Spinacia oleracea* aquaporin *PIP2;1*, SMTL id: 4jc6.2.A) [36] revealed a similarity of 78.42%, indicating that the 3D model of *KoPIP2;1* was acceptable. Homology modeling demonstrated that the 3D model structure of *KoPIP2;1* consisted of a homo-tetramer, with each monomer containing eleven *α*-helixes, two *β*-strands, some random coils, three octyl-*β*-glucanpyranoside molecules, seven cadmium ions and four mercury ions. It is suggested that the presence of cadmium and mercury ions may impact the gating mechanism of AQPs [36].

### 3.2. Phylogenic Analysis at the Protein Level

**Figure 2:**
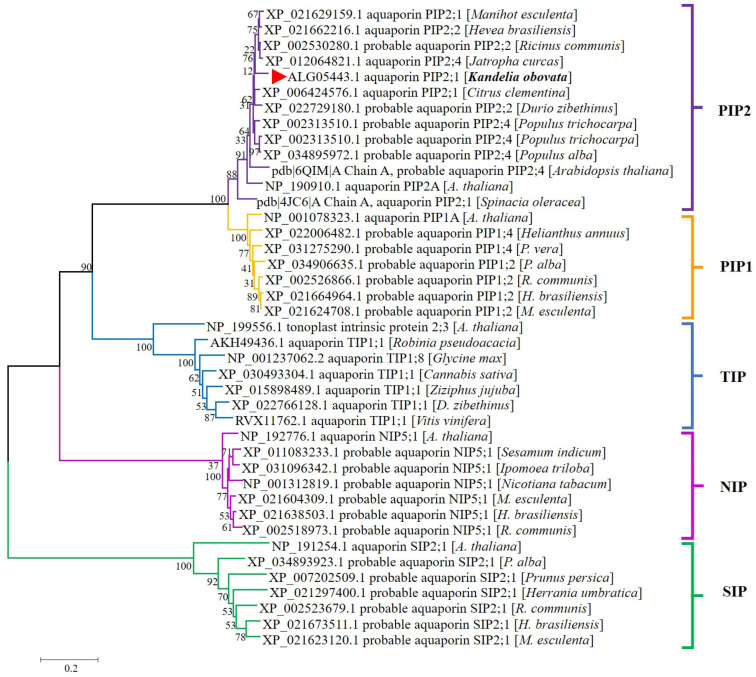
Phylogenetic relationship between *KoPIP2;1* and other plant AQPs. A total of 40 plant amino acid sequences, obtained from the NCBI database with accession numbers, were included in the tree. The *KoPIP2;1* protein is represented by a red triangle. This tree was constructed using the Neighbor-Joining method with 1000 bootstrap replications. The scale indicates the branch length.

We have downloaded representative amino acid sequences of different types of *Arabidopsis* aquaporins, as well as some aquaporins that exhibit high homology with the *KoPIP2;1* sequence through NCBI blast analysis. According to phylogenetic tree (Figure 2), *KoPIP2;1* was distinctly classified in the aquaporin PIP2 subgroup, demonstrating that *KoPIP2;1* is a member of the PIP2 subfamily. Furthermore, *KoPIP2;1* was on the same PIP2 branch as its highest homologue (*Hevea brasiliensis* aquaporin *PIP2;2*, *HbPIP2;2*) and its 3D modeling template (*Spinacia oleracea* aquaporin *PIP2;1*, *SoPIP2;1*). According to these data, we named the aquaqporin *KoPIP2;1* in this study.

### 3.3. Expression Patterns of KoPIP2;1 in Response to Cold Stress

**Figure 3:**
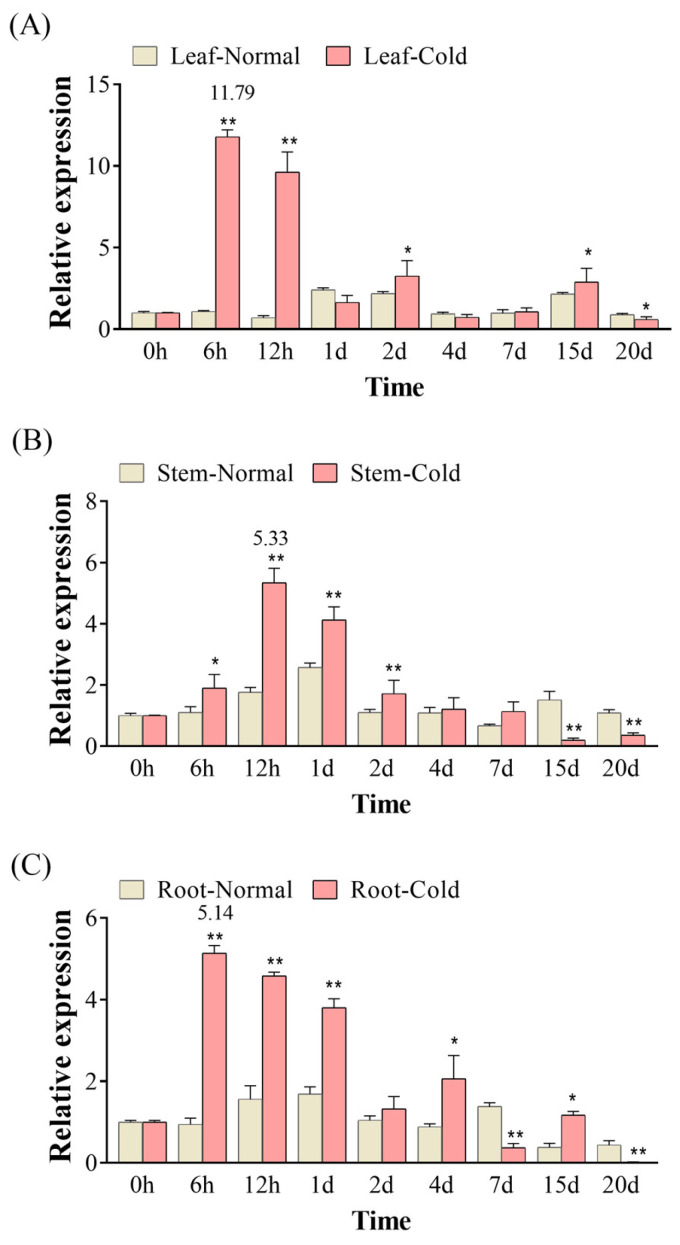
Gene expression of *KoPIP2;1* in *K. obovata* under cold stress and normal conditions. The relative gene expression levels of *KoPIP2;1* in leaf (**A**), stem (**B**) and root (**C**) were normalized using the reference gene *Ko18S*. The data represented here are the average values obtained from three biological repetitions, with error bars indicating standard deviations (*p* values were calculated using Student’s *t*-test. * *p* < 0.05; ** *p* < 0.01).

Under cold stress, the expression of *KoPIP2;1* is significantly induced in various tissues compared with normal conditions (Figure 3). The results show that the *KoPIP2;1* gene were highly expressed in the leaves, but lesser expressed in stems and roots of *K. obovata* under cold stress. During the experimental period, the expression levels of *KoPIP2;1* initially increased and then decreased in different tissues. However, the time points of their peak expression levels varied, with leaves peaking at 6 h (11.79-fold) (Figure 3A), stems at 12 h (5.33-fold) (Figure 3B), and roots at 6 h (5.14-fold) (Figure 3C). It can be stated that *KoPIP2;1* was predominantly induced during the early stage (6 h–1 d) in *K. obovata* under cold stress. Notably, significant wilting and dehydration of *K. obovata* leaves were observed after 15 days under cold stress in our previous study [37]. These results suggest that *KoPIP2;1* is an early responsive gene and may play crucial roles in the response to cold stress in *K. obovata*.

### 3.4. Subcellular Localization of KoPIP2;1 in Tobacco Epidermal Cells

**Figure 4:**
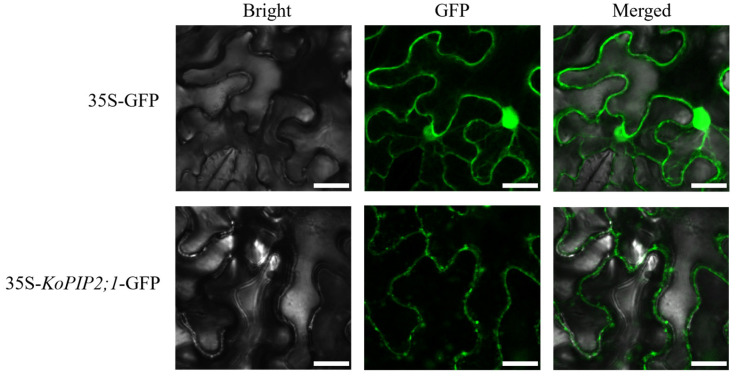
Subcellular localization of *KoPIP2;1* in *N. benthamiana* epidermal cells. GFP fluorescence was used to observe the transient expression of *KoPIP2;1*. The leaves with overexpression of 35S-*KoPIP2;1*-GFP and 35S-GFP (used as the control) were imaged by confocal microscopy. Scale bar = 100 µm.

The 35S-*KoPIP2;1*-GFP vector, labeled with fluorescence, was introduced into the epidermal cells of *N. benthamiana* and examined using confocal laser-scanning microscopy. As depicted in Figure 4, the GFP fluorescence signal of 35S-*KoPIP2;1*-GFP was predominantly accumulated in the plasma membrane, whereas the control GFP protein was distributed throughout the entire cell, including the nucleus. These findings indicate that *KoPIP2;1* is a plasma membrane protein and are consistent with bioinformatics predictions.

### 3.5. Overexpression of KoPIP2;1 Enhanced Cold Tolerance of Transgenic Arabidopsis

**Figure 5:**
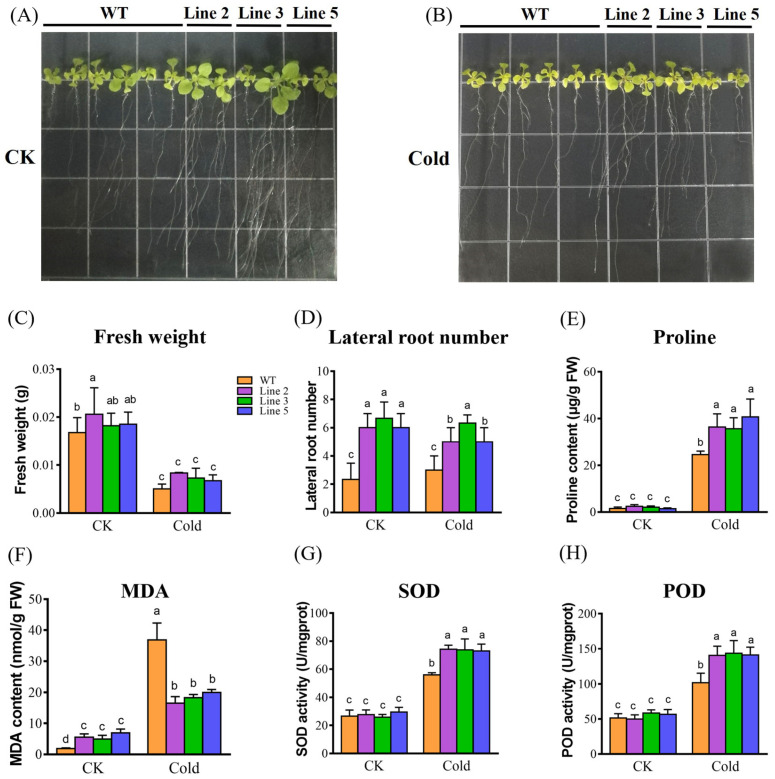
Cold tolerance analysis of *KoPIP2;1* transgenic *Arabidopsis* under cold stress. Twelve-day-old seedlings were grown in MS agar medium and subjected to normal growth condition (**A**) or cold stress condition (**B**) for 10 days, respectively. The fresh weight (**C**), lateral root number (**D**), proline content (**E**), MDA content (**F**), SOD activity (**G**), and POD activity (**H**) were measured in both the *KoPIP2;1* transgenic lines and wild-type plants. Line 2, Line 3, and Line 5 represent *KoPIP2;1* transgenic lines 2, 3 and 5, respectively. WT represents the wild type. The data were subjected to one-way analysis of variance, followed by Duncan’s test. Error bars with dissimilar letters indicate statistically significant differences (*p* < 0.05, Duncan’s test).

As shown in Figure 5, the WT *Arabidopsis* and three *KoPIP2;1* transgenic *Arabidopsis* lines (Line 2, 3, 5) showed no significant difference in fresh weight under normal growth conditions (CK) and cold stress, respectively (Figure 5A–C). Nevertheless, *KoPIP2;1* transgenic lines exhibited superior growth and significantly higher lateral root numbers than WT plants under both CK and cold conditions (Figure 5A,B,D). A well-developed root system typically results in more efficient absorption of nutrients and water, indicating that overexpression of *KoPIP2;1* could enhance the cold resistance of transgenic *A. thaliana*. To explore the involvement of *KoPIP2;1* in osmoregulation, the proline content was measured in both WT and transgenic plants. As shown in Figure 5E, transgenic lines exhibited significantly higher proline content than WT under cold stress, indicating that overexpression of *KoPIP2;1* may enhance osmoregulatory capacity by increasing the proline content of plant cells. In addition, to investigate the involvement of *KoPIP2;1* in antioxidant function, MDA content and SOD and POD activities were examined. As depicted in Figure 5F–H, the *KoPIP2;1* transgenic lines exhibited significantly lower MDA content and higher SOD and POD activities than WT plants under cold stress, indicating that overexpression of *KoPIP2;1* improved the efficiency of antioxidant systems and reduced membrane damage in transgenic *Arabidopsis* under cold stress. These findings suggest that overexpression of *KoPIP2;1* may enhance osmoregulation and antioxidant capacity in transgenic *Arabidopsis* plants, thereby conferring cold tolerance.

## 4. Discussion

The *KoPIP2;1* was located on the plasma membrane by the subcellular localization analysis, similar results of PIPs localization on the plasma membrane have been reported [38,39]. The RT-qPCR results reveal that the expression of *KoPIP2;1* exhibited tissue-specific patterns, which were also observed in other AQPs across various plant organs, including leaves, stems, roots, flowers, fruits and seeds [40]. Expression levels of most *Saussurea involucrata* PIPs were decreased in both roots and leaves under salt stress and involved in mediating water transport in tomato [41]. Tissue-specific expression of *KoPIP2;1* implies that the *KoPIP2;1* protein may play positive roles in *K. obovata*, participating in abiotic stress responses.

AQPs have been reported to mediate transcellular (across membranes) root water transport and hydraulic conductance [2]. Many PIPs have been observed to facilitate water exchange between intracellular and extracellular compartments, playing a crucial role in the maintenance of water balance within the cytoplasm [4,42,43]. Research has reported that overexpression of aquaporin genes confers benefits to transgenic plants under various stress conditions, such as cold, drought, salt, and nitrate reduction stresses [10,44,45]. In this study, it was found that the transgenic plants exhibited a higher number of lateral roots compared with WT plants under favorable conditions. This observation suggests that the *KoPIP2;1* gene has an impact on plant morphology under normal conditions in transgenic plants. Similar phenotypes have been observed in transgenic rice overexpressing *OsPIP2;3* (*Oryza sativa* PIP) and in transgenic *A. thaliana* overexpressing *PgTIP1* (*Panax ginseng* TIP) [46,47]. This is likely due to a compensation mechanism that increases lateral roots or enhanced water transport resulting from the overexpression of *KoPIP2;1*, leading to morphological changes.

Furthermore, our findings indicate that plants overexpressing *KoPIP2;1* exhibit superior growth compared with WT plants when subjected to cold stress. This suggests that *KoPIP2;1* confers tolerance to cold stress in transgenic *Arabidopsis* plants. Similarly, the overexpression of *SiPIP1;5A* (*Saussurea involucrata* PIP) enhances the cold tolerance of tomato by regulating cell water balance [48]. Conversely, some AQP members have been shown to be negatively associated with abiotic stress. The overexpression of *AtPIP1;4* and *AtPIP2;5* in plants has been seen to result in accelerated water loss during drought stress [16]. Overexpressing *AtPIP1;b* in tobacco has led to rapid wilting under drought stress [15]. Therefore, members of AQP family exhibit differential cellular functions.

Abiotic stresses always disturb osmotic balance in plants. In general, proline is widely distributed in many organisms, and the accumulation of this substance can improve cellular protection and reduce the cellular osmotic potential [49]. In this study, *KoPIP2;1* overexpressing plants exhibited higher proline level compared with WT plants, indicating that *KoPIP2;1* may regulate osmotic imbalance induced by cold stress. This finding is consistent with a previous study demonstrating that overexpression of *TsPIP1;1* (*Thellungiella salsuginea* PIP) improved cellular proline accumulation in rice under salt stress [44].

Abiotic stress induces the rapid accumulation of reactive oxygen species (ROS), which can cause severe damage to cell membranes by oxidizing DNA, proteins, and lipids [50]. MDA is commonly used to assess ROS-mediated cellular injuries and membrane damage [51]. Our findings indicate that transgenic plants exhibit lower MDA levels than the WT under cold stress, indicating that *KoPIP2;1* can mitigate membrane damage in overexpressing plants. This finding is consistent with previous studies, which have demonstrated that overexpression of *TsPIP1;1* and *MaPIP1;1* also reduces cellular MDA levels [44,52]. SOD and POD serve as ROS scavengers [53]. The overexpression of *KoPIP2;1* in plants has resulted in increased levels of both SOD and POD activities compared with WT plants. Taken together, these findings suggest that the overexpression of *KoPIP2;1* can enhance plant osmotic regulation and antioxidant capacity, which in turn reduces membrane injury. Certainly, this is only the start in terms of the study of the function of *KoPIP2;1*, further investigations, including detailed analysis in transgenic plants, are necessary to fully elucidate its role.

## 5. Conclusions

In summary, we successfully cloned and characterized the aquaporin gene *KoPIP2;1* from the mangrove plant *K. obovata.* Overexpression of *KoPIP2;1* improved the cold tolerance of transgenic *A. thaliana* by regulating solute accumulation and antioxidant capacity, suggesting an important role for *KoPIP2;1* in *K. obovata* under cold stress. Future investigations will focus on identifying upstream regulators of *KoPIP2;1* and evaluating the growth performance of these transgenic lines exposed to diverse stresses.

## Data Availability

The sequence data of *KoPIP2;1* can be obtained from the NCBI database with accession number: KP267759.1 (https://www.ncbi.nlm.nih.gov/nucleotide/KP267759.1/, accessed on 24 June 2023). All analyzed or generated data is included in this article. The data analyzed or generated in this study can be obtained from the corresponding author upon reasonable request.

## Supplementary Materials

The following supporting information can be downloaded at: https://www.mdpi.com/article/10.3390/bioengineering10070878/s1, Figure S1: Prediction of 3D structure of KoPIP2;1 and comparison with its template.
