# Supplementary material for: An Aquaporin Gene (KoPIP2;1) Isolated from Mangrove Plant Kandelia obovata Had Enhanced Cold Tolerance of Transgenic Arabidopsis thaliana
Source: Bioengineering (Basel). 2023 Jul 24;10(7):878. doi: 10.3390/bioengineering10070878 (PMC10376877; doi:10.3390/bioengineering10070878)
Supplement: Supplementary file 1 [file bioengineering-10-00878-s001.zip › bioengineering-2496218-supplementary.pdf]

bioengineering

MDPI

Supplementary Materials

An Aquaporin Gene (*KoPIP2;1*) Isolated from Mangrove Plant *Kandelia obovata* Had Enhanced Cold Tolerance of Transgenic *Arabidopsis thaliana*

Jiao Fei [1,2,3], Youshao Wang [1,2,3,*], Hao Cheng [1,2,3,*], Hui Wang [1], Meilin Wu [1,2,3], Fulin Sun [1,2,3] and Cuici Sun [1,2,3]

1 State Key Laboratory of Tropical Oceanography, South China Sea Institute of Oceanology, Chinese Academy of Sciences, Guangzhou 510301, China; feijiao@scsio.ac.cn (J.F.); wanghui224@mails.ucas.ac.cn (H.W.); mlwu@scsio.ac.cn (M.-L.W.); flsun@scsio.ac.cn (F.-L.S.); scuici@scsio.ac.cn (C.-C.S.)

2 Southern Marine Science and Engineering Guangdong Laboratory (Guangzhou), Guangzhou 511458, China

3 Innovation Academy of South China Sea Ecology and Environmental Engineering, Chinese Academy of Sciences, Guangzhou 510301, China

* Correspondence: yswang@scsio.ac.cn (Y.-S.W.); chenghao@scsio.ac.cn (H.C.); Tel.: +86-20-8902-3102 (Y.-S.W.); +86-20-8902-3132 (H.C.)

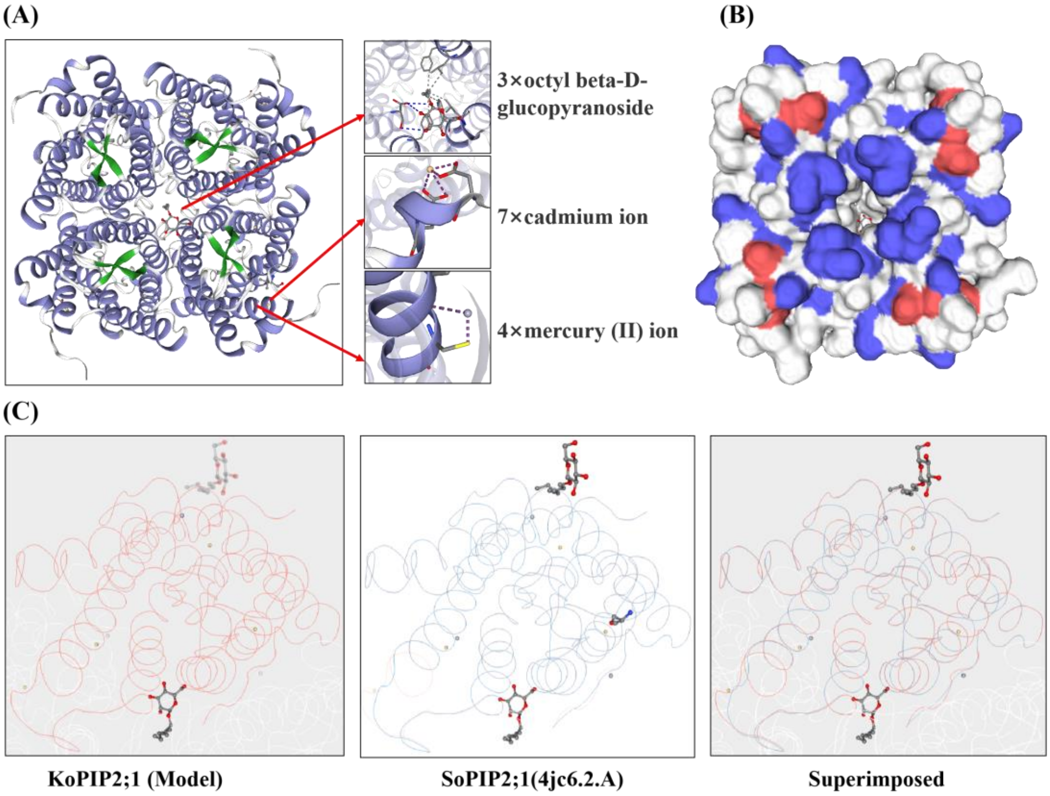

Figure S1. Prediction of 3D structure of KoPIP2;1 and comparison with its template. The 3D models of KoPIP2;1 and its template (SoPIP2;1 (*Spinacia oleracea* PIP2;1), SMTL id: 4jc6.2.A) were produced by the homology modeling SWISS-MODEL. (**A**) Left was the 3D model of KoPIP2;1 predicted ribbon structures, with α-helix (purple) and β-strand (green), random coil (white). Right was close-up view of octyl β-D-glucopyranoside-binding, cadmium-binding and mercury-binding based on homology modeling. Cadmium and mercury were shown as yellow and purple spheres, respectively. Ligands for the heavy atoms were shown in stick representation being labelled. (**B**) View of the surface topology of KoPIP2;1 showing the distribution of the electrostatic potentials. Protein surface was colored according to areas charged positively (blue), negatively (red) and neutrally (white). (**C**) Figures were predicted trace structures of KoPIP2;1 and its template SoPIP2;1 and their superimposition.
